# Natural history comparison study to assess the efficacy of elamipretide in patients with Barth syndrome

**DOI:** 10.1186/s13023-022-02469-5

**Published:** 2022-09-02

**Authors:** Brittany Hornby, William Reid Thompson, Mohammed Almuqbil, Ryan Manuel, Anthony Abbruscato, Jim Carr, Hilary J. Vernon

**Affiliations:** 1 Department of Physical Therapy, Kennedy Krieger, Baltimore, MD USA; 2 Department of Pediatric Cardiology, Taussig Heart Center, Johns Hopkins University School of Medicine, Baltimore, USA; 3 College of Medicine, King Saud Bin Abdulaziz University for Health Sciences (KSAU-HS); King Abdullah Specialized Children’s Hospital (KASCH), Riyadh, Saudi Arabia; 4 Department of Genetic Medicine, Johns Hopkins University School of Medicine, 733 N Broadway, MRB 512, Baltimore, Maryland 21205 USA; 5 Stealth BioTherapeutics, Inc, Needham, MA USA

**Keywords:** Elamipretide, Barth syndrome, Natural history control, TAZPOWER

## Abstract

**Background:**

Natural history studies are increasingly recognized as having an important role in drug development for rare diseases. A phase 3, observational, retrospective, and non-interventional study was designed to establish a natural history control (NHC) cohort of patients with Barth syndrome (BTHS) to provide further analysis of the efficacy of elamipretide observed in an open-label extension (OLE) phase of the TAZPOWER trial, a clinical trial that tested the efficacy of 40 mg daily of elamipretide in patients with BTHS.

**Methods:**

This was a retrospective, non-interventional study. A propensity score model was used to compare elamipretide-treated patients and NHCs. The analysis included 8 patients from the TAZPOWER OLE and 19 untreated NHCs (including 12 with serial echocardiographic assessments).

**Results:**

For the 6-min walk test (6MWT, primary endpoint), the least squares (LS) mean difference between groups was 79.7 m (*P* = 0.0004) at week 64 and 91.0 m (*P* = 0.0005) at week 76 in favor of elamipretide. Significant improvements in muscle strength (secondary endpoint), as assessed by handheld dynamometry (HHD) were also observed with elamipretide, with LS mean differences of 40.8 Newtons at 64 weeks (*P* = 0.0002) and 56.7 Newtons at 76 weeks (*P* = 0.0005). Patients continuously treated with elamipretide also experienced statistically significant improvements in other secondary endpoints (i.e., 5 times sit-to-stand [5XSST], multi-domain responder index [MDRI]). The functional improvements were robust to sensitivity analyses. Left ventricular stroke volume increased from baseline in patients with elamipretide but decreased in NHCs.

**Conclusions:**

Overall, the study established a NHC for use in assessing the efficacy of therapeutic interventions in patients with BTHS and the results suggest that elamipretide may improve natural history of BTHS at least in part by attenuating the natural decline in heart function and provide meaningful improvements in heart function and functional capacity in patients with BTHS compared to NHCs.

**Highlights:**

- A matched Natural History Control (NHC) was used to evaluate elamipretide in BTHS
- Elamipretide may improve natural history of BTHS by attenuating natural decline in heart function
- Elamipretide was associated with meaningful clinical improvements in skeletal muscle and cardiovascular parameters that were not observed in NHCs
- The study established a NHC for use in assessing the efficacy of therapeutic interventions in BTHS

## Introduction

Barth Syndrome (BTHS; MIM 302,060) is a rare X-linked disorder caused by defects in the gene TAFAZZIN that is characterized by multisystem involvement including: prenatal onset of left ventricular non-compaction, cardiomyopathy, intermittent neutropenia, skeletal myopathy, growth impairments and other features [1–5]. The estimated prevalence of BTHS is approximately 1 in 1,000,000 male births [6]. The characteristic biochemical findings in Barth syndrome include increased monolysocardiolipin (MLCL), decreased remodeled tetralineoyl cardiolipin (CL), and an abnormal MLCL/CL ratio in bloodspots, cells and tissues from patients [7, 8]. Increased 3-methylglutaconic acid in plasma and urine is also often seen [9]. BTHS occurs almost exclusively in males although affected females have been identified [10] [11].

Tafazzin is an acyltransferase involved in the final remodeling step of the mitochondrial phospholipid, cardiolipin (CL) [12]. CL is located in the inner mitochondrial membrane and has a central role in the maintenance and proper functioning of mitochondria, including mitochondrial protein/metabolite transport, mitochondrial morphology, and mitochondrial bioenergetics [13, 14]. Tafazzin deficiency results in decreased levels of CL and elevated levels of the structurally immature molecule, MLCL. Abnormal CL content in Tafazzin deficiency has been associated with respiratory chain dysfunction, abnormal formation of supercomplexes, and dysregulated mitochondrial quality control, among many other effects. [15–17].

Given the importance of CL in the pathophysiology of BTHS, mitochondrial targeted therapies that are CL-protective have the potential to ameliorate the underlying [15] biochemical abnormalities associated with the disease. Elamipretide is an aromatic cationic tetrapeptide that readily penetrates the mitochondrial plasma membrane and localizes to the inner mitochondrial membrane where it associates with CL. This interaction results in improved mitochondrial membrane stability and function with a normalization of energy production [18–21]. Based on these properties, elamipretide is under investigation for disorders associated with mitochondrial structural and bioenergetic abnormalities such as BTHS. However, due to the ultra-rare nature of BTHS, there are a limited number of patients available for the conduction of the conventional randomized clinical trials that are generally required to support drug registration. Elamipretide has been granted rare pediatric disease designation for the treatment of BTHS by the FDA and FDA guidance supports the use of natural history controls (NHCs) in rare disease drug development studies provided external control groups can be shown to be similar to treated groups in all respects [22].

TAZPOWER was a randomized phase 2/3 crossover trial, followed by an open-label extension (OLE) phase [23]. While no significant effect of elamipretide was observed during the 12-week randomized phase for primary clinical trial endpoints, daily subcutaneous injections of 40 mg of elamipretide were associated with significant improvements from baseline in multiple functional and cardiac outcomes during the OLE phase of the study [23]. The objective of this NHC study was to assess the effectiveness of single daily subcutaneous doses of 40 mg elamipretide as a treatment for patients with BTHS as part of the TAZPOWER study to a compared NHC cohort.

## Materials and methods

### Study design

**Fig. 1:**
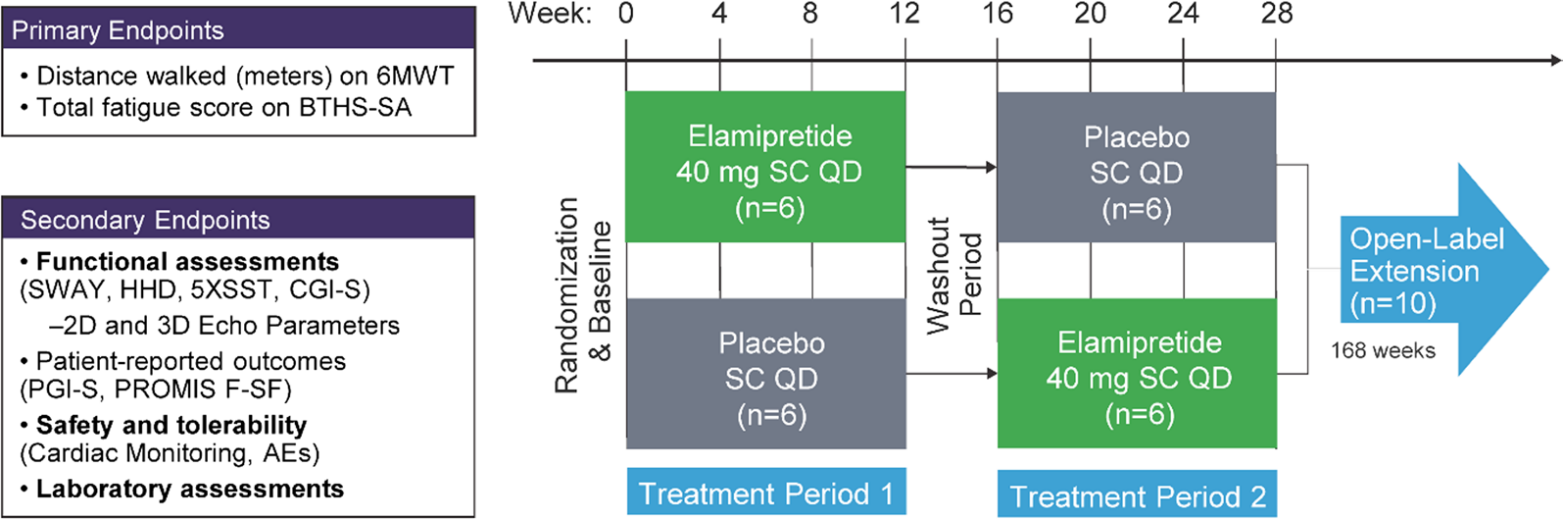
Study Design for TAZPOWER. AEs = Adverse Events; BTHS-SA = BarTH Syndrome Symptom Assessment; CGIS = Clinician Global Impression of Symptom Severity and Change Scale; 5XSST = Five Times Sit-to-Stand Test; HHD = Hand Held Dynamometry; PGI-S = Patient Global Impression of Symptom Severity and Change Scale; PROMIS F-SF = Patient-Reported Outcomes Measurement Information System Fatigue-Short Form; 6MWT = 6-Minute Walk Test; SWAY = SWAY Application Balance Assessment

This was a retrospective, non-interventional study designed to establish a NHC for valid comparison to data from TAZPOWER. The study design of TAZPOWER has been previously described [23]. In brief, TAZPOWER was a 28-week, randomized, double-blind, placebo-controlled crossover trial followed by a ≤ 168-week, open-label assessment of the long-term safety and tolerability of elamipretide in patients with genetically confirmed BTHS (Fig. 1). In the randomized phase, patients received 12 weeks of single daily subcutaneous doses of elamipretide 40 mg or placebo, followed by 12 weeks with the alternative treatment (after a 4-week washout period). In the OLE phase, patients received elamipretide 40 mg subcutaneously once daily for up to 168 weeks. The publication by Thompson et al. describing outcomes in TAZPOWER from the randomized phase of the trial and 36 weeks on OLE [23].

The TAZPOWER trial was approved under the Johns Hopkins Institutional Review Board Protocol IRB00124162 and the NHC study was approved under the Johns Hopkins Institutional Review Board Protocol IRB00231209. The trials were conducted in accordance with consensus ethics principles derived from international ethics guidelines, including the Council for International Organizations of Medical Sciences International Ethical Guidelines and the International Conference on Harmonisation Good Clinical Practice guideline. The protocol was approved by the institutional review boards of the participating trial centers and all patients provided written informed consent.

### Patients

#### Natural history controls

Natural history controls (NHCs) included patients with BTHS who attended the 2014, 2016, and 2018 Barth Syndrome Foundation International Scientific, Medical and Family Conference and participated in a clinical phenotyping assessment by our study team with no age, ethnicity or gender restrictions [24]. First-degree relatives (parents and siblings) of patients with BTHS who attended the 2016 and 2018 conferences were also eligible and were included as a normal control comparison group. In addition, individuals with a diagnosis of BTHS who attended the Barth Interdisciplinary Clinic at Kennedy Krieger Institute in Baltimore, MD for regularly scheduled follow-up care from 2014 to the present date were eligible to enroll. Many patients seen in the Kennedy Krieger Institute outpatient clinic for regular care were also assessed at the meetings (by the same study/clinical team) and therefore the data from the meetings and clinics were complementary. Approximately 80 patients were evaluated to establish an NHC for the patients who participated in TAZPOWER. All of the NHCs had molecular confirmation of a pathogenic variant in the TAFAZZIN gene and did not participate in the TAZPOWER trial.

#### TAZPOWER Inclusion/exclusion criteria

Patients ≥ 12 years of age and with genetically confirmed BTHS were eligible for inclusion in TAZPOWER. At screening, subject body weight and estimated glomerular filtration rate (eGFR) met one of the following: 1) body weight > 30 kg and eGFR ≥ 90 mL/min/1.73 m^2^ or 2) body weight > 40 kg and eGFR ≥ 60 mL/min/11.73 m^2^. Patients were required to be ambulatory and impaired (as assessed by the 6-min Walk Test [25]), on stable medications for at least 30 days prior to the study visit, and willing to use effective contraception.

Patients undergoing an apparent pubertal growth spurt, hospitalization within 30 days, uncontrolled hypertension, history of substance abuse within 1 year, history of heart transplantation or placement or on a wait list for heart transplantation (within 1 year) were excluded. Patients with an implantable cardioverter defibrillator (ICD) were excluded if there was an occurrence of ICD therapy/discharge within 3 months, as were those that were currently receiving treatment with chemotherapeutic agents or immunosuppressant agents or had received prior radiation therapy to the chest.

### Endpoints

The primary efficacy endpoint of TAZPOWER was the distance (in meters) walked during the 6MWT. Secondary efficacy endpoints included other functional tests: (1) muscle strength as measured by hand held dynamometry (HHD); (2) the time to complete the Five Times Sit-to-Stand Test (5XSST); and (3) the SWAY application balance assessment, administered to patients using the 5 stances and motion reaction test [26]. In order to characterize combined evidence associated with multiple endpoints, a patient-level multidomain responder index (MDRI) score was calculated using the primary and secondary clinical endpoints using a minimally clinically important difference of > 10% relative change from baseline.

Exploratory three-dimensional echocardiographic measurements included left ventricular (LV) end-diastolic, LV end-systolic, and LV stroke volumes (mL), all indexed to body surface area (BSA), as well as LV ejection fraction.

Natural history data were entered into a single-center, clinical site-managed electronic data capture system (REDCap®). To ensure consistency with respect to assessment methodology and conduct, all assessments were captured using standard protocols and in a manner similar to that of the TAZPOWER trial by the same clinical team. As this was an ongoing prospective observational, natural history study, there was no set frequency with which the data was collected; the data was captured when patients were seen in clinic for regular care and/or attended the 2014, 2016 and 2018 conferences.

### Statistical analysis

A propensity score model was used to derive stabilized weights based on inverse probability of treatment weighting methods to balance the two cohorts with respect to important baseline covariates. In order to avoid selection bias for the NHC subjects, the statisticians who developed the propensity model only had access to the baseline TAZPOWER data. In order to balance the two cohorts and further minimize the impact of selection bias on estimates of treatment differences, logistic regression was used to compute propensity scores and stabilized weights for eligible patients in the TAZPOWER and NHC cohorts using age, height- and baseline 6MWT distance walked as baseline prognostic covariates. For the efficacy analyses, NHCs were compared to the post-TAZPOWER baseline data in treated patients. The impact of selection bias on differences between groups was minimized by using a common set of cohort eligibility criteria and propensity score methods.

Sensitivity analyses were performed to assess the robustness of the observed functionality results. These analyses assessed additional timepoints (sensitivity analysis 1), an alternative propensity model (sensitivity analysis 2), unweighted results for the main analysis (sensitivity analysis 3), and the treatment effects for discontinued patients (sensitivity analysis 4).

For the exploratory 3-dimentional echocardiographic analysis, LV stroke volume was indexed to baseline BSA. Cardiovascular imaging endpoints were evaluated using two approaches: a slope model analysis and a mixed model analysis. In the slope model, the slopes of stroke volume over time for each patient were estimated using ordinary least squares and compared to the slopes for NHCs using a one-way analysis of variance. The mixed model repeated measures analysis approach was performed for change from baseline, where baseline for NHCs was the first available time point. Left ventricular end diastolic volume index (LVEDVI) and left ventricular end systolic volume index (LVESVI) were analyzed via a similar methodology.

## Results

### Patients

Overall, there were 12 patients from the TAZPOWER and 79 subjects with data from the REDCap® database evaluated for inclusion, which includes 10 total patients who continued on to the OLE phase of the study. The primary analysis includes 8 patients from the TAZPOWER OLE who reached week 72 in the OLE, and 19 untreated NHCs who had non-missing baseline prognostic covariate data and at least one post-baseline non-missing data for the primary endpoint. Baseline prognostic covariates for NHCs and elamipretide-treated patients from TAZPOWER are summarized in Table 1. Mean age (18.3 vs 21.0 years), height (166.6 vs 168.6 cm), and baseline 6MWT (381.9 vs 394.9 m) were generally similar between the two populations. For the cardiovascular analysis, there were 12 patients treated with elamipretide and 12 from the NHC who had at least two serial echocardiogram assessments.

**Table 1:** Baseline prognostic covariates

Characteristic	TAZPOWER patients (*n* = 8)	Natural history controls (*n* = 19)
Age at baseline, years		
Mean (SD)	18.3 (5.02)	21.0 (5.46)
Range	12.9 –28.7	12.0 – 32.6
Height at baseline, cm		
Mean (SD)	166.6 (12.43)	168.6 (14.25)
Range	152.5 – 180.8	121.3 – 186.0
Baseline 6MWT		
Mean (SD)	381.875 (64.1837)	394.879 (75.2197)
Range	313.00 –495.00	267.00 –536.45

6MWT = 6-min walk test, *SD *Standard deviation

### Exposure and data collection

The overall mean (standard deviation [SD]) duration of exposure to elamipretide treatment in both the randomized and open-label extension phase among those in the TAZPOWER group was 116.1 (9.99) weeks. The overall duration of data collection for functionality endpoints is summarized in Table 2. Duration of data collection ranged from 75 to 196 weeks for the NHC for various endpoints and was 113 weeks for all endpoints among those receiving elamipretide in TAZPOWER.

**Table 2:** Overall duration *of data collection for efficacy endpoints

	Duration of data collection in weeks, mean (SD)	
	TAZPOWER Patients (*n* = 8)	Natural history controls (*n* = 19)
6MWT	113.1 (12.59)	195.9 (65.09)
Muscle Strength by HHD	113.1 (12.59)	175.0 (46.82)
5XSST	113.1 (12.59)	83.3 (45.81)
SWAY	113.1 (12.59)	74.9 (50.58)^a^

^a^*n* = 18

*6MWT* 6-min walk test, *HHD *handheld dynamometry; *5XSST *5 times sit-to-stand, SD = standard deviation

^*^Duration of data collection ranged from 75 to 196 weeks for the NHC for different endpoints and was 113 weeks for all endpoints for those receiving elamipretide in TAZPOWER

### Functional assessments

Changes from baseline in functional outcomes at weeks 64 and 76 are summarized in Table 3. For the primary efficacy endpoint (6MWT), patients who received continuous treatment with elamipretide experienced a significantly greater increase from baseline to week 64 and to week 76 compared with the NHCs. The least squares (LS) mean difference between groups was 79.703 m (*P* = 0.0004) at week 64 and 90.972 m (*P* = 0.0005) at week 76. The sensitivity analyses supported the results of the main analysis with all comparisons showing statistically significant improvements in 6MWT at all time points assessed.

**Table 3:** Change from baseline in functional endpoints

Mean (SD) change from baseline	TAZPOWER patients (*n* = 8)	Natural history controls (*n* = 19)
**6MWT, meters**		
LS mean change from baseline at **Week 64**	80.299	0.596
LS mean difference (95% CI); *P* value	79.703 (40.46, 118.95); *P* = 0.0004	
LS mean change from baseline at **Week 76**	91.858	0.886
LS mean difference (95% CI); *P* value	90.972 (44.57, 137.38); *P* = 0.0005	
**Muscle strength by HHD**		
LS mean change from baseline at **Week 64**	41.789	1.035
LS mean difference (95% CI); *P* value	40.755 (21.70, 5980); *P* = 0.0002	
LS mean change from baseline at **Week 76**	48.667	1.970
LS mean difference (95% CI); *P* value	40.755 (21.70, 59.80); *P* = 0.0005	
**5XSST**		
LS Mean change from baseline at **Week 64**	− 2.361	− 0.002
LS mean difference (95% CI); *P* value	− 2.359 (− 4.62, − 0.10); *P* = 0.0416	
LS mean change from baseline at **Week 76**	− 2.829	− 0.003
LS mean difference (95% CI); *P* value	− 2.825 (− 5.41, − 0.24): *P* = 0.0340	
**SWAY application balance assessment**		
LS Mean change from baseline at Week 64	7.398	0.862
LS mean difference (95% CI); *P* value	6.536 (− 2.21, 15.28); *P* = 0.1312	
LS mean change from baseline at Week 76	8.806	1.084
LS mean difference (95% CI); *P* value	7.722 (− 2.40, 17.84); *P* = 0.1240	
**MDRI**		
LS Mean change from baseline at Week 64	3.013	0.634
LS mean difference (95% CI); *P* value	2.379 (1.34, 3.42); *P* = 0.0001	
LS mean change from baseline at Week 76	3.119	0.709
LS mean difference (95% CI); *P* value	2.411 (1.38, 3.44); *P* = 0.0001	

*6MWT* 6-min walk test, *HHD *Handheld dynamometry, *MDRI *Multi-domain responder index, *5XSST* 5 times sit-to-stand, *SD* Standard deviation

Patients continuously treated with elamipretide also had improvements in secondary functional endpoints (HDD, 5XSST, SWAY application Balance, and MDRI) at weeks 64 and 76. The differences between elamipretide-treated patients and NHCs were statistically significant for muscle strength by HDD (LS mean differences = 40.755 Newtons at 64 weeks; *P* = 0.0002 and 56.697 Newtons at 76 weeks; *P* = 0.0005; 5XSST (LS mean differences =  − 2.361 s at 64 weeks; *P* = 0.0416 and − 2.829 s at 72 weeks; *P* = 0.0340), and MDRI (LS mean differences = 3.013 at 64 weeks; *P* = 0.0001; and 3.119 at 72 weeks; *P* = 0.0001). As for the primary endpoint, the results were robust to sensitivity analysis. Differences between treatment groups in the SWAY Application Balance Assessment did not reach statistical significance.

### Cardiovascular imaging endpoints

For the exploratory 3-dimentional echocardiographic analysis, 12 NHCs were compared to 12 TAZPOWER participants who completed the placebo-controlled portion of the clinical trial and had at least 12 weeks of exposure to elamipretide. LV stroke volume index (LVSVI) increased significantly in patients treated with elamipretide compared with untreated NHCs when using either the slope model (*P* = 0.0407) or the mixed model analyses (*P* = 0.0020) (Table 4). In the slope model, there was a 3.44 (5.26) mL mean (SD) increase in LVSVI from baseline to study endpoint in the elamipretide group compared with a 0.26 (2.66) mL decline for untreated controls (*P* = 0.0407). Similarly, when using the mixed model analysis, the improvement in LVSVI at week 100 for patients receiving elamipretide (+ 1.92 mL) was significantly greater than what is expected in the natural course of the disease (− 4.8 mL) over a comparable time period (*P* = 0.002). There were also treatment differences in LVEDVI and LVESVI using both the slope model and the mixed model analyses; however, these differences were not significant.

**Table 4:** Change from baseline in cardiovascular imaging endpoints*

	TAZPOWER Patients (*n* = 12)		Natural History Controls (*n* = 12)	
*Slope Model Analysis*				
	LV stroke volume (mL)			
		Mean (SD) change from baseline; range	3.44 (5.26); − 1.7, 18.3	− 0.26 (2.66); − 4.9, 3.7
		*P* value	0.0407	
	LV end-diastolic volume (mL)			
		Mean (SD) change from baseline; range	4.89 (7.25); − 1.5, 24.7	0.91 (6.15); − 12.3, 11.2
		*P* value	0.1609	
	LV end-systolic volume (mL)			
		Mean (SD) change from baseline; range	1.46 (2.26); − 2.0, 6.4	1.17 (4.29); − 7.4, 9.1
		*P* value	0.8417	
*Mixed Model Analysis*				
	LV stroke volume (mL)			
		LS mean change from baseline	1.92 − 4.80	
		LS mean difference (95% CI), *P* value	6.72 (2.75, 10.69); *P* = 0.0020	
	LV end-diastolic volume (mL)			
		LS mean change from baseline	2.08 − 2.66	
		LS mean difference (95% CI), *P* value	4.75 (− 2.72, 12.22); *P* = 0.2006	
	LV end-systolic volume (mL)			
		LS mean change from baseline	0.52 1.28	
		LS mean difference (95% CI), *P* value	− 0.77 (− 7.13, 5.60); *P* = 0.8047	

*Indexed to baseline body surface area

*CI* Confidence interval, *LS* Least squares, *LV* left ventricular, *SD* standard deviation

## Discussion

There are unique challenges associated with clinical trial design and drug development in ultra-rare conditions such as BTHS. These challenges may include limited longitudinal natural history data to inform clinical trial design, incomplete understanding of the effects of allelic heterogeneity, and limited participant pools available to power successful clinical trials.

Natural history studies, defined as a preplanned observational studies intended to track the course of a disease, have historically been used to understand the clinical characteristics and disease progression in under characterized diseases. More recently, natural history studies have been increasingly used to facilitate drug development, particularly in rare diseases [22]. The important role of natural history studies in drug development has become more formalized with the publishing of draft industry guidance by the FDA [22]. In particular, the guidelines allow for the use of natural history data as an external control group in clinical drug development. Current guidance states that NHCs can be useful as a comparator to a treated group provided the two groups are similar with respect to disease characteristics and history (eg, disease severity, duration of illness, prior treatments, etc.) that could affect outcomes and the timing of outcomes [22]. The use of external NHCs is especially useful where there are no available alternative treatments and where an internal control (ie, placebo) is not appropriate. As a result, natural history modeling for orphan drug development is receiving increased attention and such analyses have been performed for a number of rare diseases.[27].

The current study was designed to establish a control group to discriminate treatment effects from other influences including temporal clinical variation in the natural history of the disease. Given the low prevalence of BTHS syndrome, conducting additional adequate and well-controlled clinical trials would be extremely challenging due to lack of available/eligible study participants. Thus, in accordance with FDA draft guidance, we pursued a study to support efficacy observations in the open-label portion of TAZPOWER through detailed analysis of a NHC.

The current analysis compares functional outcomes of patients receiving elamipretide to that of a matched NHC cohort over a prolonged time period. The results indicate a treatment benefit of long-term elamipretide therapy in functional endpoints compared to NHC who did not receive elamipretide. Specifically, we observed significant improvements in 6MWT, HDD, 5XSST, and MDRI compared to those observed in the NHC cohort. In the cardiovascular imaging analysis, elamipretide was also associated with significant improvements in LVSVI compared with the untreated NHC group, which may coincide with observed improvements in functional domain outcomes. These functional and cardiovascular improvements are clinically relevant and likely to improve patients’ quality of life. For example, we found that patients receiving elamipretide achieved a 79.7 m greater improvement in the 6MWT compared with those in the NHC group. Studies in various disease states have estimated that the minimum clinically important difference for improvement in the 6MWT ranges from 25 to 35 m [28–30]. Importantly, the results were supported by sensitivity analyses.

BTHS most often presents with cardiomyopathy, which may be a dilated, hypertrophic, LVNC, or mixed phenotype, exercise intolerance, muscle weakness and severe fatigue that negatively impact QoL, growth failure/delay, and intermittent neutropenia [31]. Stroke volume, and therefore cardiac output, is often low in subjects with BTHS despite apparent normal contractile function, [32] and, in agreement with these prior observations, stroke volume experienced a gradual decline in the NHCs in the current study and elamipretide use was associated with increases in left ventricular stroke volume. The effect on stroke volume is likely the result of an increase in both left ventricular end systolic (LVESV) and left ventricular end diastolic volumes (LVEDV) but with LVEDV increasing to a greater amount than LVESV. We hypothesize that the augmentation of LVSV in this population is due to relatively small left ventricular volumes at baseline. Importantly, there were increases in left ventricular mass observed in these patients, which may indicate that the favorable changes in LVEDV are both structural and functional. It is not clear whether increase in LV chamber size and stroke volume may be, at least in part, due to improvements in skeletal muscle function and exercise ability.

The limitations of the study include its observational nature and the limited number of patients available. However, this analysis demonstrates the power of longitudinal natural history data in ultra-rare diseases in evaluating and validating clinical trial outcomes. Furthermore, propensity matching of baseline characteristics for NHC/TAZPOWER participants could not account for all clinical and management variations in individual medical care between patients including prescribed medications, frequency of medical assessments, etc.

In summary, this study established a NHC that is valuable for assessing the efficacy of therapeutic interventions in patients with BTHS. The results suggest that long-term therapy with elamipretide may improve natural history of BTHS by attenuating the natural decline in heart function and provide meaningful improvements in heart function and functional capacity in patients with BTHS compared with NHC.

## Data Availability

Please contact author for data requests.
